# Complete Genome Sequences of Two Gammaproteobacterial Methanotrophs Isolated from a Mercury-Contaminated Stream

**DOI:** 10.1128/MRA.00181-21

**Published:** 2021-05-20

**Authors:** Christina S. Kang-Yun, Jin Chang, Scott C. Brooks, Baohua Gu, Jeremy D. Semrau

**Affiliations:** a Department of Civil and Environmental Engineering, University of Michigan, Ann Arbor, Michigan, USA; b Environmental Sciences Division, Oak Ridge National Laboratory, Oak Ridge, Tennessee, USA; University of Southern California

## Abstract

The genomes of *Methylomonas* sp. strain EFPC1 and *Methylococcus* sp. strain EFPC2, isolated from a mercury-contaminated stream in Oak Ridge, Tennessee, were sequenced.

## Abstract (precis)

The genomes of *Methylomonas* sp. strain EFPC1 and *Methylococcus* sp. strain EFPC2 isolated from a mercury-contaminated stream in Oak Ridge Tennessee, were sequenced.

## ANNOUNCEMENT

Two methanotrophs of the *Gammaproteobacteria* class, *Methylomonas* sp. strain EFPC1 and *Methylococcus* sp. strain EFPC2, were isolated from the mercury-contaminated East Fork Poplar Creek (EFPC) in Oak Ridge, Tennessee, from biofilm samples collected in July, 2020 (specific sampling locations, N35.990385°, W84.317983° and N35.992482°, W84.315327° for *Methylomonas* sp. EFPC1 and *Methylococcus* sp. EFPC2, respectively). Biofilm samples were first inoculated in nitrate mineral salts medium (1) in liquid culture at 30°C with methane as the sole carbon and energy source to enrich for methanotrophs. After visible growth on methane, samples were then streaked onto NMS agar plates as described earlier (2). After repeated streaking onto NMS plates with purity confirmed via microscopy, 16S rRNA gene sequencing and negative growth on nutrient agar plates (3), a single colony of each strain was then grown in NMS liquid medium with methane. DNA from 50-ml and 200-ml cultures were extracted using phenol-chloroform extraction (4) and Qiagen Genomic-tip 500/G (Qiagen, Hilden, Germany) for Illumina and GridION Nanopore sequencing, respectively. Libraries for Illumina sequencing were prepared using a NEBNext Ultra II FS DNA Library Prep Kit (New England Biolabs, Inc., Ipswich, MA) with 15-min fragmentation and size selected for 275 to 475 bp. Libraries for GridION Nanopore sequencing were prepared using ligation sequencing and native barcoding expansion kits (SQK-LSK109 and EXP-NBD104; Oxford Nanopore Technologies, Littlemore, UK) following the manufacturers’ protocols. Genomic DNA (gDNA) was sequenced using separate Nano flow cells and 500 cycle V2 kits on a MiSeq sequencer (Illumina, Inc., San Diego, CA) at the University of Michigan Advanced Genomics Core (AGC). Long-read sequencing was performed on the GridION X5 platform at the University of Michigan AGC (Oxford Nanopore Technologies, Littlemore, UK). Basecalling was performed using Guppy (v.4.2.3) (5). Sequence quality was assessed using FastQC (v0.11.9) (6) before and after trimming. The short and long reads were trimmed using Trimmomatic (v0.39) (7) and Porechop (v0.2.4) (8), respectively, and then were assembled using Unicycler (v0.4.9b) with no correction (9). Assembly completeness was assessed via BUSCO (v4.1.4) (10) and also visually confirmed using Bandage (v0.8.1) (11). The final contigs were annotated using the National Center for Biotechnology Information Prokaryotic Genome Annotation Pipeline (v5.1) (12). The annotated 16S rRNA sequences were used as queries in search of the most similar organism using the Basic Local Alignment Search Tool (BLAST; v2.11.0) (13). Default parameters were used for all software unless otherwise specified.

*Methylomonas* sp. strain EFPC1 and *Methylococcus* sp. strain EFPC2 genomes were 4.99 Mbp and 4.56 Mbp (96% and 95.2% completion), consisting of either 1 chromosome and 1 plasmid (for *Methylomonas* sp. strain EFPC1) or 1 chromosome and 2 plasmids (for *Methylococcus* sp. strain EFPC2). All chromosomes and plasmids were circularized and then rotated according to the starting gene via Unicycler and were visually inspected using Bandage. 16S rRNA sequence analyses of *Methylomonas* sp. strain EFPC1 indicated that it was phylogenetically similar to *Methylomonas* sp. LW13 (14), and *Methylococcus* sp. strain EFPC2 was most similar to *Methylococcus geothermalis* IM1^T^ (15). Average nucleotide identity (ANI) values between *Methylomonas* sp. strain EFPC1 and *Methylomonas* sp. LW13 and between *Methylococcus* sp. strain EFPC2 and *Methylococcus* sp. IM1^T^ were ∼95% and 73%, respectively (16). Genes for particulate methane monooxygenase (pMMO) were found in both *Methylomonas* sp. strain EFPC1 and *Methylococcus* sp. strain EFPC2, while evidence of a divergent form of pMMO (pXMO) and soluble methane monooxygenase was found in only *Methylomonas* sp. strain EFPC1. These results are summarized in Table 1.

**TABLE 1:** General features of the *Methylomonas* sp. strain EFPC1 and *Methylococcus* sp. strain EFPC2 genomes

Strain	Complete genome size (bp)	No. of plasmids	G+C content (%)	Total no. of coding sequences	No. of rRNA genes (16S, 23S, and 5S)	No. of tRNA genes	Methane monooxygenase(s) present	No. of Illumina reads (accession no.)	No. of GridION reads (accession no.)	*N*_50_ of GridION reads (bp)	Annotated genome sequence accession no.	Closest neighbor based on 16S rRNA sequence	16S rRNA similarity with closest neighbor (%)	ANI^*a*^ with closest neighbor (%)
*Methylomonas* sp. strain EFPC1	4,993,755	1	51.8	4,488	9	47	pMMO, pXMO, sMMO	2,230,538 (SRX10121820)	232,890 (SRX10121821)	51,070	CP070494, CP070495	*Methylomonas* sp. LW13	99.87	95.20
*Methylococcus* sp. strain EFPC2	4,558,902	2	61.2	3,891	9	50	pMMO	2,379,682 (SRX10121822)	135,052 (SRX10121823)	50,620	CP070491, CP070492, CP070493	*Methylococcus geothermalis* IM1^T^	96.30	72.70

a Average nucleotide identity.

### Data availability.

Accession numbers for the annotated sequences and raw reads are posted in Table 1.
